# Large-Dynamic-Range Ocular Aberration Measurement Based on Deep Learning with a Shack–Hartmann Wavefront Sensor

**DOI:** 10.3390/s24092728

**Published:** 2024-04-25

**Authors:** Haobo Zhang, Junlei Zhao, Hao Chen, Zitao Zhang, Chun Yin, Shengqian Wang

**Affiliations:** 1 National Laboratory on Adaptive Optics, Chengdu 610209, China; 2 School of Automation Engineering, University of Electronic Science and Technology of China, Chengdu 611731, China; 3 Institute of Optics and Electronics, Chinese Academy of Sciences, Chengdu 610209, China; 4 University of Chinese Academy of Sciences, Beijing 100049, China; 5 Eye School, Chengdu University of TCM, Chengdu 610075, China; 6 Key Laboratory of Sichuan Province Ophthalmopathy Prevention & Cure and Visual Function Protection with TCM, Chengdu 610075, China

**Keywords:** dynamic range, wavefront sensing, ocular aberration measurement, Shack–Hartmann wavefront sensor, deep learning, wavefront reconstruction

## Abstract

The Shack–Hartmann wavefront sensor (SHWFS) is widely utilized for ocular aberration measurement. However, large ocular aberrations caused by individual differences can easily make the spot move out of the range of the corresponding sub-aperture in SHWFS, rendering the traditional centroiding method ineffective. This study applied a novel convolutional neural network (CNN) model to wavefront sensing for large dynamic ocular aberration measurement. The simulation results demonstrate that, compared to the modal method, the dynamic range of our method for main low-order aberrations in ocular system is increased by 1.86 to 43.88 times in variety. Meanwhile, the proposed method also has the best measurement accuracy, and the statistical root mean square (RMS) of the residual wavefronts is 0.0082 ± 0.0185 λ (mean ± standard deviation). The proposed method generally has a higher accuracy while having a similar or even better dynamic range as compared to traditional large-dynamic schemes. On the other hand, compared with recently developed deep learning methods, the proposed method has a much larger dynamic range and better measurement accuracy.

## 1. Introduction

There are a variety of low-order (defocus and astigmatism) and high-order (except defocus and astigmatism) optical aberrations in human eyes that change dynamically with time, significantly reducing the resolution of retinal imaging. In addition, ocular aberration also has individual differences. The SHWFS has become a widely used tool for measuring eye aberrations in ophthalmic applications due to the simplicity and versatility. The SHWFS has two performance characteristics, sensitivity and dynamic range, and there is a clear tradeoff between them [1]. Due to the dispersion of the population and the large fluctuation of aberration amplitude [2], it is difficult to reliably measure some eyes with large aberrations based on SHWFS, especially for eyes with large pupil size since the dynamic range is usually insufficient [3]. Because of the excessive aberration, the wavefront fluctuation is serious, which will cause the spot to deviate from the original corresponding sub-aperture. As a result, it is difficult to measure the aberration accurately since the traditional wavefront reconstruction methods (modal algorithm [4] and zonal algorithm [5,6,7]) will fail.

There are many methods for expanding the dynamic range [8,9,10,11,12,13,14,15,16,17,18,19,20,21,22,23,24,25], and the most significant advantages of software-based approaches are excellent flexibility and low cost, since they do not require special hardware. The current software-based methods are mainly based on using extrapolation to match the centroid with the corresponding sub-aperture more precisely to improve the dynamic range of detection. Pfund, J. proposed that each spot is trapped in a naturally assigned sub-aperture, and their method spread out the spot displacement, that is, assigned the spot to the correct reference sub-aperture. The limitation of this method is that the spot deviation cannot exceed half of the distance P between sub-apertures [8]. In order to assign spots to their reference points, an iterative spline extrapolation method was proposed by Groening [10]. Leroux designed an extrapolation method based on Zernike polynomials [13], but this method was susceptible to the initial point. The method was proposed by Smith et al., assuming that the positions of the spots are arranged in a predetermined order; then, it uses local spot position distortion to unfold the spot pattern, but this method will not work if some spots are missing in the Hartmannogram [12]. Yu, L. et al. proposed a Gaussian correlation matching algorithm to find the center of spots, which improves the dynamic range of different terms of the Zernike polynomial by 57.1∼160% [15]. However, if some spots are too weak to be seen or detected, their method will not work. Gao used the Canny operator to segment each spot from the Hartmannogram, and then matched the spots using the neighborhood search algorithm, which improves the dynamic range by 64.7∼205.4%. This method is effective even if there are more than two spots in one sub-aperture, but the starting point of the method must be located in the correct sub-aperture region [16]. Chen integrated the above two methods, proposed a new Gaussian correlation to find the center of spots, and then optimized the neighborhood search algorithm to pair spots [17]. His method achieved a greater improvement in the dynamic range. However, when the aberration is overly large, the shape of the spot no longer follows the Gaussian model, and the proposed method cannot find or accurately locate the spot, leading to large errors. The method proposed by Yang Wen et al. is not an extrapolation method, but the core idea is also to match spots with the sub-aperture. The matching is implemented using a neural network after autocorrelation computation to extract the spot centroid from the Hartmannogram, improving the dynamic range of the Zernike polynomial by 62.86∼183.87% [18]. The essence of all these methods is to match the spots with the corresponding sub-apertures and reconstruct the wavefront via the modal algorithm, leading to their shortcomings. Moreover, they are unable to address issues such as spot overlap and cross-talk, and their effectiveness can be easily influenced by the initial point.

In addition, other scholars have proposed alternative methods. For instance, Roggemann. M.C. proposed the method of optimizing traditional image intensity and Hartmannogram composition functions (with the Zernike coefficient as the independent variable) [9]. Although the method has a high accuracy, the constructed function cannot converge every time, and the method takes a long time because it is too complex. The technique proposed by Lee divides the spot array into rows and columns, but this method does not work when point and row intersections occur [11]. Vargas proposed a regular pyramid LK optical flow iteration algorithm [14]. This algorithm is available in harsh environments, but the speed is limited due to the complexity of the process.

The deep learning methods proposed by some scholars can avoid the error caused by the sub-aperture matching problem in principle, but they are not intended to expand the dynamic range. In 2006, Guo et al. [26] combined a neural network and SHWFS for the first time and proposed a method to obtain the Zernike coefficient of aberration based on SHWFS spot displacement by using an artificial neural network. Inspired by this approach, numerous approaches combining deep learning with SHWFS have emerged in recent years. In 2018, Li et al. [27] proposed a SHWFS centroid calculation method (SHNN) for AO systems with strong ambient light and noise pollution from the perspective of improving centroid positioning accuracy. The method uses a three-layer fully connected neural network to transform the spot detection problem into a classification problem. The input is the intensity of 625 pixels of the sub-aperture image, and the output uses 625 nodes to represent all these categories. Inspired by [28], in 2020 Hu et al. [29] proposed a method to obtain a phase map directly from the SHWFS spot pattern, called SH-Net. This method is based on the improved ResUnet [30]. In 2022, Guo et al. [31] proposed a simple but effective network for phase retrieval, reaching a root mean square error (RMSE) of 0.0360.

Almost all of the above methods are applied in astronomy using atmospheric turbulence models. The distribution of aberrations in the field of the human eye is different from them. The spatial characteristics of ocular aberrations are mainly manifested in the dispersion of population and the large fluctuation of the amplitude in low-order aberrations. The amplitude of higher-order aberrations is small, but the spatial frequency is high [2]. An aberration in the field of astronomy is a typical zero-mean Zernike pattern combined aberration, which is mainly consistent with Kolmogorov turbulence [32]. Meanwhile, none of them are committed to solving the dynamic range problem of SHWFS. In this paper, a deep learning approach is utilized for a large range of ocular aberration detection while maintaining high accuracy. Unlike the tradeoff dilemma, this method improves the measurement accuracy and dynamic range at the same time. In addition, due to the characteristics of deep learning, this method can directly learn the end-to-end mapping between Hartmannograms and reconstructed wavefronts. This mapping is represented by a trained deep CNN that is effective on both normal and abnormal Hartmannograms. The abnormal Hartmannograms are defined as some spots that exceed the original sub-aperture range or shift out of the imaging sensor plane.

The structure of the paper is as follows. In Section 2, the formation of Hartmannograms, will be introduced; the simulation process and the network structure are given in Section 3. Then, the experimental results and dynamic range analysis are given in Section 4. Finally, the conclusion and future work outlooks are presented in Section 5.

## 2. Basic Principles of SHWFS

**Figure 1:**
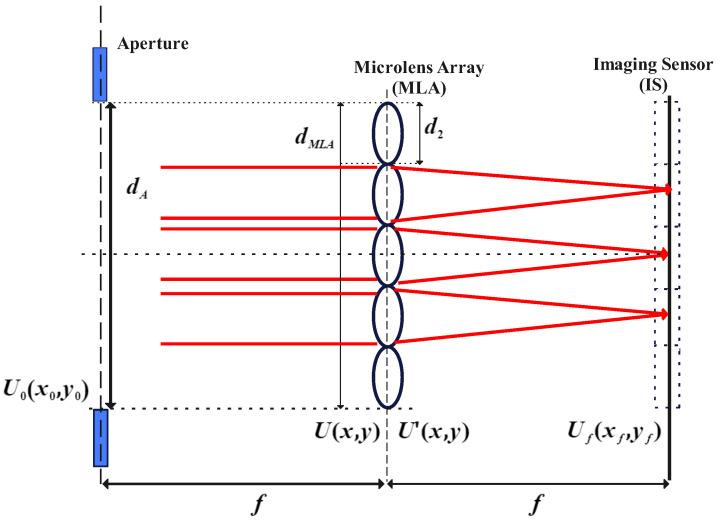
SHWFS Structure. Red lines represent the light ray.

The structure of SHWFS is shown in Figure 1. It is composed of an aperture, a micro lens array (MLA), and an image sensor (IS). The size of aperture, denoted by $d_{A}$, is equivalent to that of the MLA. $d_{MLA}$ is the length of MLA, and $d_{2}$ is the length of a lenslet. The aperture and IS are placed in the front focal plane and the back focal plane, respectively, and the focal length is denoted by *f*.

We assume that the phase of the complex amplitude (CA) of the incident wavefront on the entrance pupil is given by

(1) $$W\left(x_{0},y_{0}\right)=\sum_{j=1}^{K}a_{j}Z_{j}\left(x_{0},y_{0}\right)$$

where $a_{j}$ is the jth coefficient, and $Z_{j}\left(x_{0},y_{0}\right)$ is a modal function like that of Zernike modes with a maximal order *K*.

Then, the CA at the aperture is

(2) $$U_{0}\left(x_{0},y_{0}\right)=A\cdot \exp\left(jW\left(x_{0},y_{0}\right)\right)P_{AM}\left(x_{0},y_{0}\right)$$

where *A* is the amplitude and $P_{A}M\left(x_{0},y_{0}\right)$ is the pupil function of the aperture.

Then, the CA at the front plane of MLA is represented by

(3) $$U\left(x,y\right)=\frac{1}{j\lambda f}\exp(jkf\exp\left[j\frac{k}{2f}\left(x^{2}+y^{2}\right)\right]F{\left\{U\left(x_{0},y_{0}\right)\exp\left[j\frac{k}{2f}\left(x_{0}^{2}+y_{0}^{2}\right)\right]\right\}}_{f_{x}=\frac{x}{\lambda f},f_{y}=\frac{y}{\lambda f}}$$

where F is the Fourier transform operator, and k=2π/λ is the wave number.

Then, the CA at the back plane of MLA can be derived with

(4) $$U^{\prime }\left(x,y\right)=U\left(x,y\right)M\left(x,y\right)\exp\left[-j\frac{k}{2f}\left(x^{2}+y^{2}\right)\right]$$

where M(x,y) is the phase mask function.

Lastly, the phase map in the plane of IS is computed by

(5) $$U_{f}\left(x_{f},y_{f}\right)=\frac{1}{j\lambda f}\exp\left[j\frac{k}{2f}\left(x_{f}^{2}+y_{f}^{2}\right)\right]F{\left\{U^{\prime }\left(x,y\right)\exp\left[j\frac{k}{2f}\left(x^{2}+y^{2}\right)\right]\right\}}_{f_{x}=\frac{x_{f}}{\lambda f^{\prime }},f_{y}=\frac{y_{f}}{\lambda f}}$$

where $\left(x_{f},y_{f}\right)$ is the coordinate of the sensor plane.

The quadratic phase bending has no effect on the intensity when recording and measuring the intensity distribution in the plane of observation

(6) $$I_{f}\left(x_{f},y_{f}\right)={\left(\frac{1}{\lambda f}\right)}^{2}{\left|F{\left\{U^{\prime }\left(x,y\right)\exp\left[j\frac{k}{2f}\left(x^{2}+y^{2}\right)\right]\right\}}_{f_{x}=\frac{x_{f}}{\lambda f^{\prime }}f_{y}=\frac{y_{f}}{\lambda f}}\right|}^{2}$$

## 3. Simulation

### 3.1. Neural Network Model

**Figure 2:**
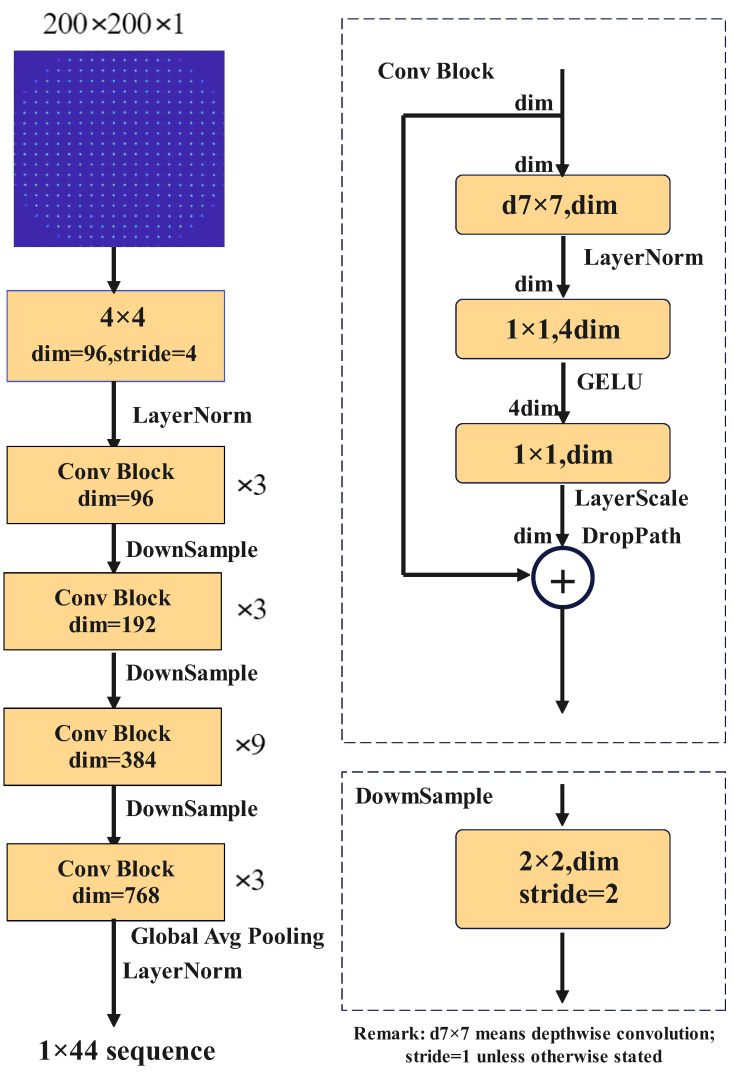
Sketched map of the LDROAD network for wavefront reconstruction. It has convolution blocks and downsample blocks.

A convolutional neural network is utilized in our experiment, and is also termed as the Large Dynamic Range Ocular Aberration Detection Convolution Neural Network (LDROAD). The LDROAD stems from ConvNeXt [33], shown in Figure 2. In detail, we change the input size and output channels of the original model to transfer a classification task into a regression task. The ConvNeXt starts from a ResNet-50 model [34], which adjusts a series of design decisions including stage ratio, patchify stem, depth, inverted bottleneck, large kernel size, activation function, normalization settings, and so on. For the task of classification, the ConvNeXt model can outperform the Swin Transformer which is utilized in wavefront reconstruction. Thus, the ConvNeXt model has the potential to surpass the Swin Transformer in a wavefront reconstruction task. There are four different variants ConvNeXt-T/S/B/L which have the same architectures but different channels and corresponding batches to be of similar complexities to Swin-T/S/B/L. In our implementation, the LDROAD references ConvNeXt-tiny as the main model due to the adequate number of parameters. Four highlights may make LDROAD perform so excellently. Firstly, the blocks of convolution layers in LDROAD are stacked in a 3:3:9:3 ratio, different from the 3:4:6:3 ratio in ResNet-50. The second is that the convolution block consists of a depthwise convolution layer [35] and two deeper pointwise convolution layers, which provides accuracy benefits. Moreover, the inverted bottleneck structure is applied in the network, which means the dimension in the middle is higher than the two ends. Finally, a larger convolution kernel in the convolution block is used from 3 × 3 to 7 × 7. The last layer is an output fully connected layer whose channel is determined by the desired prediction channel. In our experiment, the number of the output channel is 44.

### 3.2. Loss Function and Evaluation Index

As for the loss function, the kernel of root mean square error is both a loss function and an evaluation index. The loss function is Equation (7) and the evaluation function is Equation (8).

(7) $$\;\mathrm{MSELoss}\;=\sum_{i=1}\sqrt{\sum_{j=1}^{K}{\left(x_{\mathrm{pred}\;,i,j}-x_{\mathrm{true}\;,i,j}\right)}^{2}}$$

(8) $$RMSE=\frac{1}{N}\sum_{i=1}^{N}\sqrt{\sum_{j=1}^{K}{\left(x_{pred,i,j}-x_{\mathrm{true}\;,i,j}\right)}^{2}}$$

where $x_{pred,i,j}$ represents the j-th Zernike coefficient of the i-th prediction sample reconstructed from our model and $x_{true,i,j}$ represents that of the label sample which is the ground truth. K is the number of Zernike terms and N is the number of samples inputting neural networks, K=44 in this paper. Thus, after summing the j subscript, a phase map is obtained. Then, summing the i subscript can obtain the loss and averaging the summation can obtain the evaluation value.

### 3.3. Simulation Process

**Figure 3:**
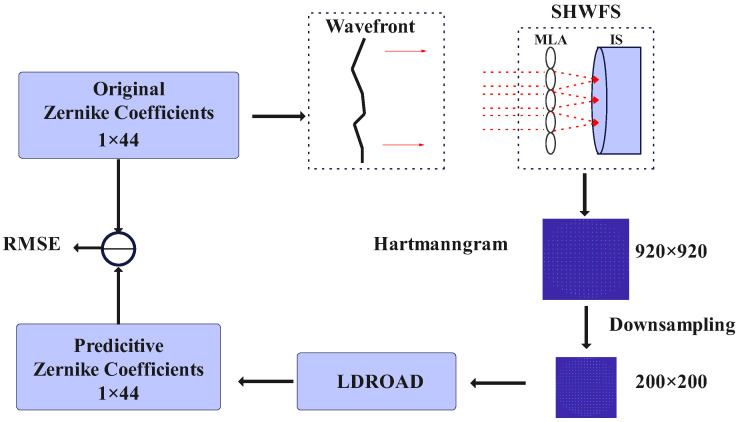
Flowchart. Red dashed line represent the light rays.

A flowchart that illustrates how the process works is shown in Figure 3. The process is divided into four stages.

In the first stage, the used wavefront is generated by a set of Zernike coefficients. Each wavefront has the characteristic of ocular aberrations.

At the processing stage, there are several steps remaining. Firstly, the wavefront $W(x_{0},y_{0})$ starts from the source and is expressed as $U(x_{0},y_{0})$ at the aperture. Then, the wavefront will be transmitted to the front plane of MLA. The process can be regarded as the Fresnel diffraction. From the front plane to the back plane, the complex amplitude of the wavefront should multiply the quadratic phase. After passing through the lens, the process will repeat from the back plane of MLA to the focal plane. There is an image sensor (usually CCD) located at the focal plane. Photons hit the sensor to form light spots and the spot pattern is called a Hartmannogram.

At the third stage, the Hartmannogram whose size is 920×920 is downsampled into 200×200 and 224×224 for the sake of computational efficiency and memory. These three versions have the same corresponding Zernike coefficients. All of them are saved, 200×200 for use in the LDROAD and SHCNN, 224×224 for use in the SHNet, and the other one for conventional methods.

At the final stage, the downsampled Hartmann is transferred to the LDROAD, and a new sequence of Zernike coefficients is obtained. RMSELoss and RMSE are computed from the new sequence of Zernike coefficients and original sequence of Zernike coefficients for updating the neural network and display performance, respectively.

The whole process returns to the starting point to form a closed loop, which can effectively verify the predicted results.

### 3.4. Simulation Setup

#### 3.4.1. Parameters

To prove that neural networks can extend the detection range, synthetic data artificially created based on the Fourier optics (as described in Section 2) is used. The detailed parameters of the SHWFS are almost referenced to [36,37], shown in Table 1.

#### 3.4.2. Simulated Dataset

The human eye data used in this paper are from an ocular aberration generator created by Xiao Fei, a graduated student from our research group [38]. This generator references Thibos et al. [39] to describe ocular aberrations. The generator derived from actual 332 normal and 344 disease eyes human eye aberration data establishes a statistical model of human eye aberrations conforming to the characteristics of Chinese human eye aberrations.

For the two types of human eyes, the Gaussian model can better fit each Zernike term, and a chi-squared test (*p* = 0.05) indicates that 37 of the 44 Zernike aberrations in the first eight orders can be fitted well using the Gaussian model. Therefore, human eye aberrations can be described by the multivariate Gaussian model, where the mean and variance are analyzed from the converted Zernike coefficients.

(9) $$y=f\left(x,\mu ,\Sigma \right)=\frac{1}{\sqrt{\left|\Sigma \right|}{\left(2\pi \right)}^{d}}e^{-\frac{1}{2}{\left(x-\mu \right)}^{T}\Sigma^{-1}\left(x-\mu \right)}$$

where μ and Σ represent the mean and covariance matrix of vector *x*, respectively.

In addition, compared with the actual measured aberration, the model can describe the aberration characteristics of normal and diseased human eyes well through the statistical analysis.

#### 3.4.3. Number of Dataset

The Hartmannograms are synthetically created from simulated sets of Zernike coefficients based on the Fourier optics as described in Section 2. There are 24,000 samples for training, 8000 samples for validating, and 8000 samples for testing. The whole dataset is approximately divided into three parts in a 6:2:2 ratio. Additionally, the set of samples has three versions. The input sample whose size is 200×200 is used in the LDROAD for enhancement of computation efficiency and memory use reduction. The sample whose size is 920×920 is used in conventional methods. The input size for SHCNN is also 200×200, but the input size of SHNet is 224×224 due to the special network structure. Therefore, the size of the results shown in the example section is 224×224. Both the 200×200 and 224×224 samples are obtained from the 920×920 image downsampling. The downsampling is realized by bicubic interpolation. The only difference between the training set, the validation set, and the test set is the input size; everything else is the same.

#### 3.4.4. Training Details

The order does not matter in the training phase, and in the validation phase, the order is shuffled for greater universality and performance. The batch size of this experiment is eight, and the number of works is also eight. The optimizer for LDROAD is Adam with an initial learning rate of $3\times 10^{-4}$, and the weight decay is set as 0.05. As an essential hyperparameter in supervised learning and deep learning, the learning rate determines whether the objective function can converge to the local minimum and when it converges to the minimum. A suitable learning rate can make the objective function converge to the local minimum in a suitable time. The learning schedule is taken as a cosine descent with an end factor of $1\times 10^{-6}$ for different initial learning rates. When we use the stochastic gradient descent algorithm to optimize the objective function, the learning rate should become smaller as the value becomes closer to the global minimum of the loss value to make the model as close to this point as possible, and cosine annealing can reduce the learning rate by using the cosine function. In the cosine function, as *x* increases, the cosine decreases slowly first, then accelerates, and then decreases slowly again. This drop pattern can be combined with the learning rate in a highly efficient way to produce decent results. The progress of the cosine descent depends on the number of epochs; the number of epochs is 500 for the LDROAD model under different conditions. Other details such as strategy and settings are the same. In addition, both SHCNN and SHNet train in accordance with the relevant settings described above.

The training, validation, and testing were performed on a deep learning workstation with Intel Core-9-11900K CPU (Intel, Santa Clara, CA, USA) and NVIDIA GTX 3090 (Nvidia, Santa Clara, CA, USA) using the Pytorch 18.04 architecture.

## 4. Result

### 4.1. Loss Result

**Figure 4:**
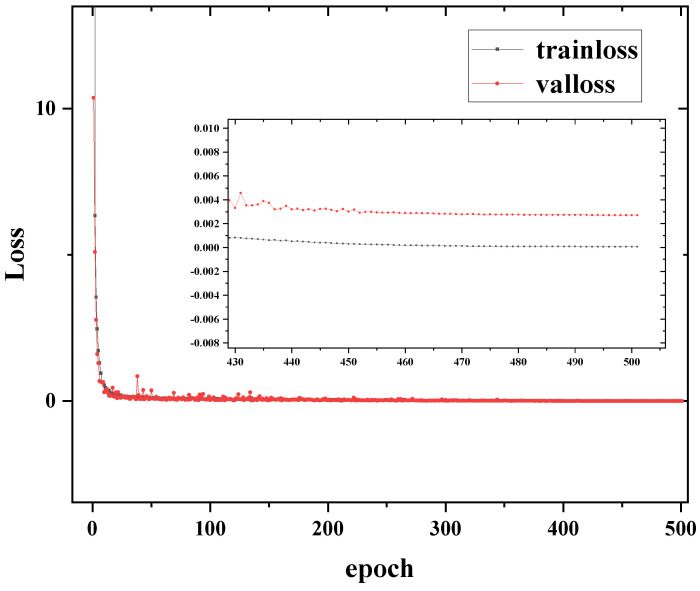
Training and validation loss curve of the LDROAD during the training process.

Figure 4 shows the training process when the initial learning rate is 0.0003. In addition, Figure 4 also shows a detailed view from epoch = 430 to the end of training. The loss of training and validation starts from 433.4748 and 10.3652, respectively. However, both of them decrease quickly. When the epoch reaches 7 and 6, the training loss and validation loss are less than 1, respectively. When the epoch reaches 54 and 31, the training loss and validation loss are less than 0.1, respectively. Finally, the training loss stays at $7.4936\times 10^{-5}$ and the loss of validation is $2.7129\times 10^{-3}$.

### 4.2. Result of Example Display

**Figure 5:**
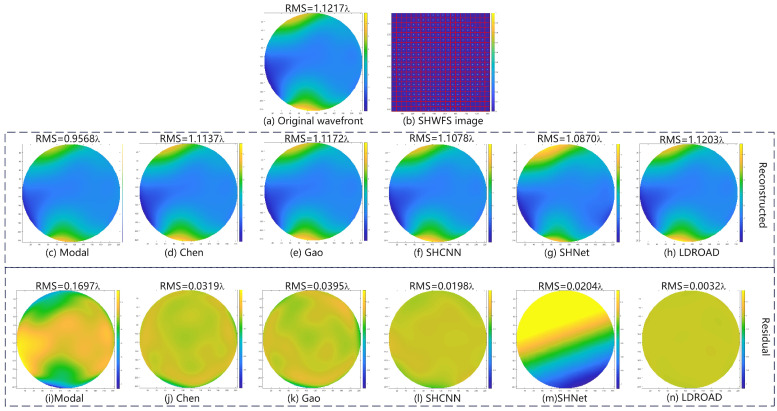
Normal example. (**a**) Original wavefront, (**b**) SHWFS image, (**c**–**h**) are the reconstructed wavefront, and (**i**–**n**) are the residual wavefront, generated by the modal, Chen’s, Gao’s, SHCNN, SHNet, and LDROAD methods, respectively.

**Figure 6:**
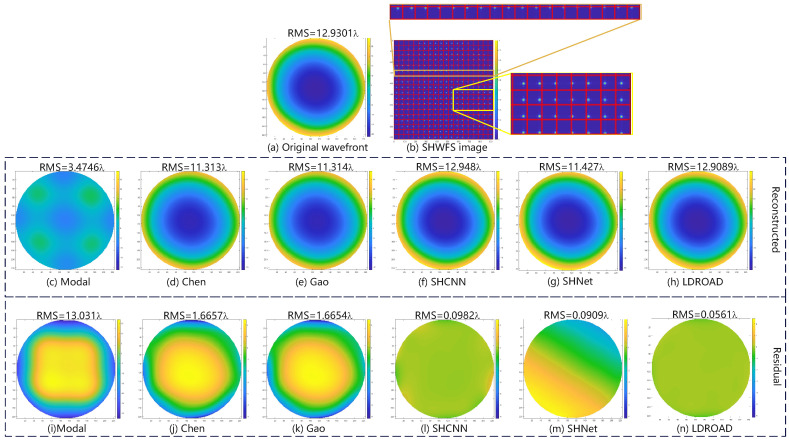
Abnormal example. (**a**) Original wavefront, (**b**) SHWFS image, (**c**–**h**) are the reconstructed wavefront, and (**i**–**n**) are the residual wavefront, generated by the Modal, Chen’s, Gao’s, SHCNN, SHNet, and LDROAD methods, respectively.

In our experiment, there are two typical examples, including normal examples and abnormal examples, where abnormal examples are the spots that exceed the boundary or disappear in the Hartmannogram. Thus, two representative samples are shown in Figure 5 and Figure 6, including an input wavefront, a corresponding spot pattern, a predictive wavefront, and a residual phase map. Note that the red grids simply indicate for observers and readers whether spots move out of own sub-apertures, and in actuality, our process unit only receives Hartmannogram without red grids.

For the normal sample, the RMS of the input wavefront is 1.1217λ, and it is not a strong disturbance, so a regular Hartmannogram is obtained. Although the difference between the predicted wavefront and the input wavefront is not visible to the naked eye, it can be seen from the residual wavefront whose RMS is 0.0032λ. The RMS of the residual wavefront is 0.0319λ and 0.0395λ for Chen’s and Gao’s method, respectively. The result of SHCNN is 0.0198λ, while that of SHNet is 0.0204λ. The reconstructed wavefront has an obvious shift. The modal algorithm is also basically reconstructed to the similar waveform of the input wavefront. But the accuracy of our method is considerably higher than that of other methods.

For the abnormal sample, the RMS of the input wavefront is 12.9301λ and the aberration is so large that some spots move out of the imaging sensor and there are only 18 spots in a row. Under this harsh circumstance, the RMS of the predictive wavefront is 12.9089λ, closing to the input RMS, and the RMS of the residual phase map is 0.00561λ, and the residual wavefront RMS values are 1.6657λ, 1.6654λ, 0.0982λ, and 0.0909λ for Chen’s, Gao’s, SHCNN, and SHNet methods, respectively. Improved methods (Chen’s and Gao’s methods) and other deep learning methods can reconstruct the basic wavefronts but the errors are too large in some conditions (greater than 1/14λ). The conventional modal algorithm fails to reconstruct the wavefront in such a harsh condition. The wavefront reconstructed using the modal method completely loses the basic appearance and characteristics. From this comparison, we can find that the LDROAD still works successfully in harsh conditions, and the strong aberration and the disappearance of spots will not affect the effect of wavefront reconstruction.

### 4.3. Comparison of Test Dataset

Table 2 shows the statistical results of six methods. The original RMS (without tilt-tip) of 8000 sets of Zernike mode coefficients in the test set of the LDROAD is 5.8244±2.9831λ. The estimated RMS of 8000 sets of the phase map (without tilt-tip) predicted by the LDROAD is 5.8243±2.9830λ. The most important index in this experiment is the RMSE of the LDROAD which is 0.0082±0.0185λ. The RMSE of Chen and Gao’s method is 0.4626±0.3942λ and 0.5019±0.3941λ, respectively. The conventional modal algorithm can only obtain 3.2753±3.4655λ. Two deep learning methods also achieve good results, with the SHCNN having an error of 0.0227±0.0371λ and SHNet having an error of 0.0251±0.0227λ. The reconstructed wavefronts at least look very similar for the other three methods under normal circumstances. However, the two improved methods and other deep learning methods are greatly affected, and their errors increase in abnormal situations. The conventional method is completely invalid, and the reconstructed wavefront is completely deformed. In contrast, LDROAD works for most scenarios.

**Figure 7:**
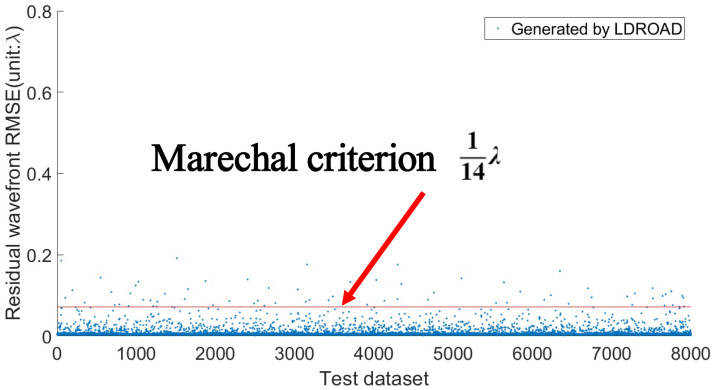
The residual wavefront RMSE results of 8000 test data sets. The red line is a Marechal criterion line, below which the reconstructed wavefront meets the requirement.

Figure 7 shows the RMSE predicted by the LDROAD for each sample. The red line is a Marechal criterion [40] line (1/14λ), below which the wavefront recovery meets the requirement (Marechal criterion). After performing the statistical analyses, more than 7900 samples of the 8000 samples meet the requirements, and the reconstruction rate of LDROAD is as high as 98.99%. Not only is the mean very low, but it is also easy for individuals to meet the requirement. The RMSE values of some samples are high because some Zernike terms exceed the ability of LDROAD. Alternatively, it is probably because too many spots are missing or shift out of the imaging sensor plane due to tremendous aberrations, leading to less than 70% of valid information remaining in the Hartmannogram.

### 4.4. Dynamic Range Analysis

**Figure 8:**
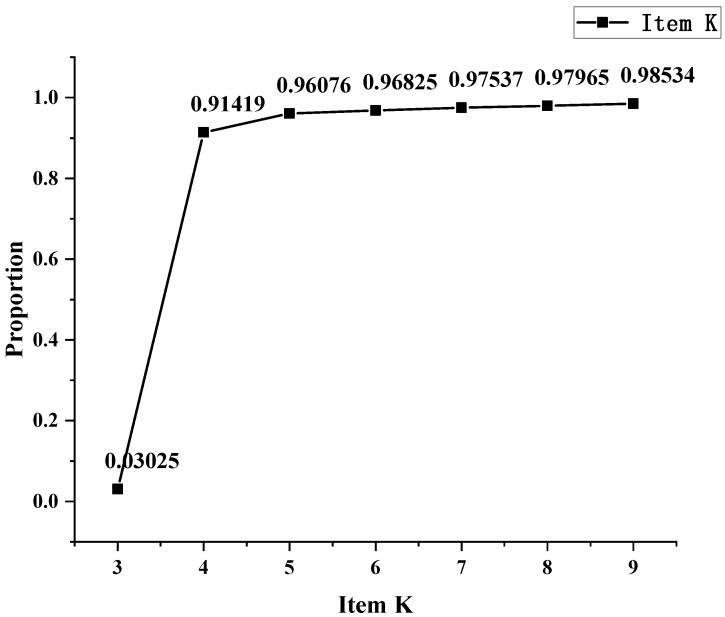
Sum of first K items’ proportions.

In order to explore how many Zernike items are to be tested, firstly, we calculate the square of each sample’s item in the test set and the sum of 44 Zernike items, denoted as $z_{i,j}$ and $z_{t,j}=\sum_{i=3}^{44}z_{i,j}$, respectively, where *i* represents the corresponding Zernike item and *j* represents the sample index. The second step is to calculate the first K items and the proportion $p_{K,j}$ for each sample, namely, $p_{K,j}=\frac{\sum_{i=3}^{K}z_{i,j}}{z_{t,j}}$. Finally, it is reasonable to obtain the mean of $p_{K,j}$ under different K values (Figure 8). The first three orders of aberrations account for 98.5% of the total aberrations, so we will focus on analyzing the dynamic range of low-order ($Z_{3}-Z_{9}$) aberrations.

Due to the uninterpretable nature of deep learning, the method in this study is not explained by mathematical formulas like traditional methods. Therefore, it is difficult to define its dynamic range using the relevant mathematical formulas. Zernike polynomials are orthogonal and linearly independent of each other, and the coefficients of Zernike polynomials are positively correlated with RMS. In view of these properties, the dynamic range can be measured by increasing the coefficients of the Zernike polynomials when the other terms of coefficients remain zero. Although the dynamic range means that the spot does not exceed the sub-aperture, if the RMS of the recovered residual wavefront does not exceed the threshold, it is considered that the dynamic range is not exceeded. The threshold is 0.0894 in this experiment, which gives a 25% tolerance.

The order of the test data is from 1 to 3 $\left(Z_{3}∼Z_{9}\right)$ and aberration details are listed in Table 3. Firstly, the detection is determined in a large range. There are some critical values that the RMS of the residual wavefront will only just satisfy the requirement. In other words, if some values are a little smaller (or greater) than the critical values in the negative direction (in the positive direction), the RMS of the residual wavefront of the values will exceed the threshold. The improvement in dynamic range is computed as Equation (10). Then, the detection range is gradually narrowed down around the critical values until the accuracy reaches one thousandth.

(10) $$\delta_{DR}=\frac{CV_{LDROAD}-CV_{modal}}{CV_{modal}}$$

**Figure 9:**
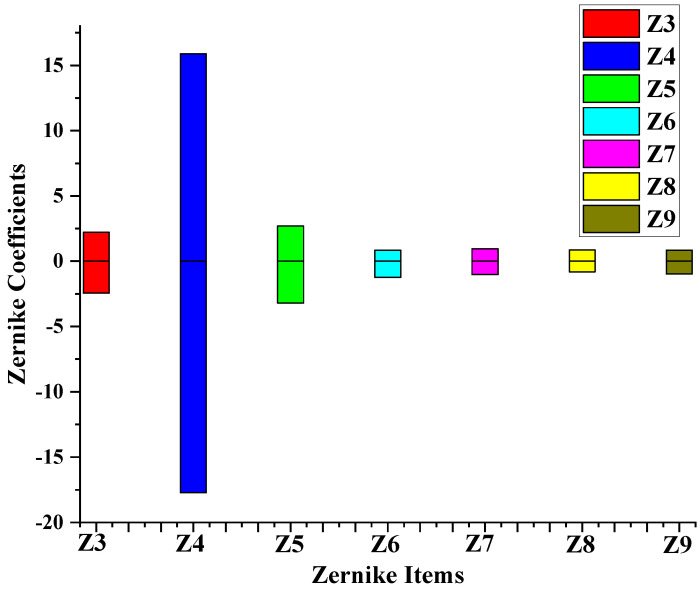
Distribution of test aberrations.

The range of tested data is from μ−3σ to μ+3σ, where μ is the mean and σ is the standard deviation. Each coefficient fits the distribution law of the Gaussian model, so the range of three times deviation should cover all possible samples (See Figure 9).

**Figure 10:**
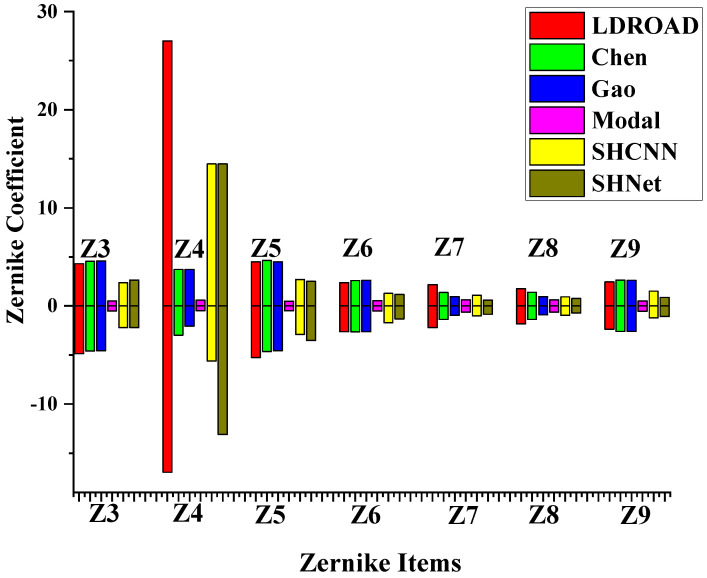
Comparison of the dynamic range in the SHWFS for different aberrations.

**Figure 11:**
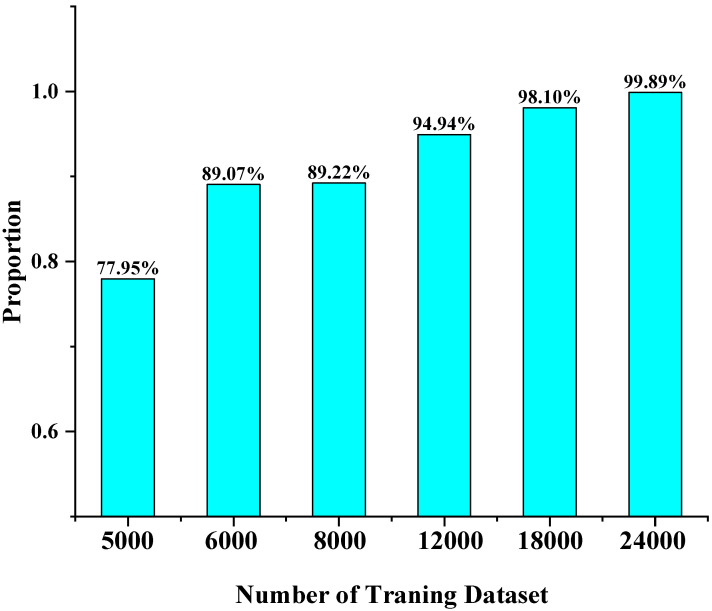
Training on a limited amount of data. The horizontal coordinate is the number of training samples. The vertical coordinate is the proportion of the number of samples in the test set that meet the Marechal criterion.

Figure 10 shows the distribution of tested Zernike items from the test dataset. Figure 11 provides the results for the dynamic ranges of the six methods, with the same color representing the same method and the columns next to each other representing the same Zernike term. All improved methods and deep learning methods are superior to the traditional modal method. Generally, the LDROAD has a slightly better performance in the negative value region and a similar performance in the positive region when compared with Gao’s and Chen’s method. The most significant change is the defocus term (Z4), the main reason for the spot running out of the sub-aperture. This phenomenon satisfies the distribution of ocular aberrations and Zhao’s conclusion in [2]. Both deep learning methods exceed improved methods in the defocus term but worse than the improved methods and LDROAD in other items. In most cases, the SHCNN outperforms the SHNet, which is also verified in the statistical results. The largest value of the defocus term of the LDROAD can obtain 27.0189 and −16.9396 in the positive and negative region, respectively. Compared with the traditional modal algorithm, the dynamic range of LDROAD for such aberrations is improved by 186.08% to 4388.19%.

### 4.5. Limited Data

To analyze the performance of the LDROAD model under a limited amount of training data, we conducted corresponding experiments to explore the relationship between the amount of LDROAD training set data and the model performance, as shown in Figure 11 and Table 4. The numbers of training sets include 5000, 6000, 8000, 12,000, and 18,000. The training model, learning rate, learning strategy, and number of epochs are the same as the formal training, and the training samples are randomly selected from the formal training set. Their test sets are the same as the official test, which is 8000 samples. As can be seen from the figure, more than 80% of the samples can reach the Marechal criterion when the minimum number of training data is 6000.

### 4.6. Generalization Test

**Figure 12:**
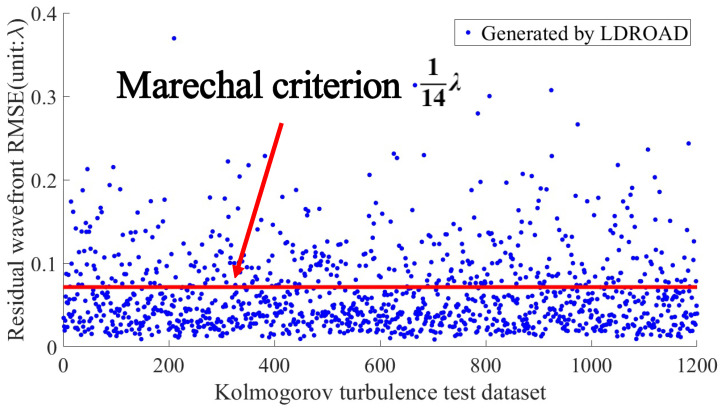
Generalization test. The RMSE of the test data set is 0.0640λ.

The analysis of LDROAD’s dynamic range in this paper proves its generalization performance to some extent. In this experiment, the Zernike pattern distribution in the dynamic range test is completely different from the Zernike pattern distribution in the LDROAD test. The data in the test set are consistent with the characteristics of the human eye, while the data in the dynamic range test are single-mode. In addition, to analyze the generalization ability of LDROAD, 1200 samples were generated according to the statistical characteristics of Kolmogorov turbulence [32] to verify the generalization of the model. Instead of retraining the model, we used the weight 0.0082λ to test the generalization. The results are shown in Figure 12. It can be seen from the figure that even for data subject to Kolmogorov turbulence characteristics, LDROAD can still reconstruct the wavefront very accurately, and the residual RMSE of the wavefront is 0.0640λ, close to the Marechal criterion.

## 5. Conclusions

In this paper, the state of research and the challenges with the traditional method of Shack–Hartmann wavefront measurement are reviewed. These algorithms only compute the centroid position of the spot in the corresponding pixel region, which is the sub-aperture of each micro-lens. Therefore, if a spot is outside the sub-aperture, the reconstructed wavefront will not be correct. This is also often the case in human eye wavefront detection. In this research, the neural network model LDROAD is applied in ocular wavefront detection carried out in a larger range with higher accuracy. We also compare the performance of the classical method, the improved methods, and deep learning methods applied in ocular wavefront measurement. Under normal conditions where the spot is located in a sub-aperture, the estimation accuracy of this method is much higher than that of other methods. Our approach allows the Hartmannogam to lose some information within a certain range. Under extreme conditions, that is, when the spot exceeds the sub-aperture, the spot exceeds the imaging sensor, and spots disappear, our method still works successfully and the performance of this method is significantly better than that of the classical method and other methods. In conclusion, the experimental results show that the dynamic ranges of the first nine Zernike polynomials such as defocus, astigmatism, coma, high-order coma, and spherical aberrations are significantly improved using this method, especially the defocus term. Moreover, the method we proposed can accurately measure the primary and higher aberrations in almost all cases in the human eye domain because the experiment results demonstrate that the RMS values of nearly 99% of the residual wavefronts meet the Marechal criterion. Additionally, the proposed method has achieved excellent results on the generalization ability test and the limited amount test, which also meet the Marechal criterion.

The proposed method obtains good performance, but it still has some restrictions. Accurate reconstruction of wavefronts in other areas will require more comprehensive data to feed the network in order to realize its true potential. In addition, the LDROAD can obtain such good performance because of its deep layers, which also has negative effects on the computation efficiency. In the future, we will conduct further studies to resolve the limitations. Deploying the LDROAD on a real machine is our next task.

## Figures and Tables

**Table 1:** Parameters of SHWFS used in the simulation setup.

Parameters	Value
Diameter D(mm)	6
Wavelength(nm)	840
MLA size	20×20
Size of each microlens (um)	300
Pixel size (um)	6.5217
Focal length of MLA (mm)	15
Sub-aperture size (pixel)	46×46

**Table 2:** Comparison of six methods.

Method	Modal	Chen	Gao	SHCNN	SHNet	LDROAD
Original RMS	5.8244±2.9831λ	5.8244±2.9831λ	5.8244±2.9831λ	5.8244±2.9831λ	5.8244±2.9831λ	5.8244±2.9831λ
Estimated RMS	3.4970±1.1937λ	5.5027±2.8183λ	5.4741±2.4679λ	5.8233±2.9828λ	5.2193±2.5721λ	5.8242±2.9835λ
RMSE	3.2753±3.4655λ	0.4626±0.3942λ	0.5019±0.3941λ	0.0227±0.0371λ	0.0251±0.0227λ	0.0082±0.0185λ

**Table 3:** Equations of different Zernike terms and corresponding aberrations.

Zernike Polynomials	Aberration
$Z_{3}=\sqrt{6}\rho^{2}\sin2\theta$, $Z_{5}=\sqrt{6}\rho^{2}\cos2\theta$	Astigmatism
$Z_{4}=\sqrt{3}\left(\rho^{2}-1\right)$	Defocus
$Z_{6}=\sqrt{8}\rho^{3}\sin3\theta$, $Z_{9}=\sqrt{8}\rho^{3}\cos3\theta$	Coma
$Z_{7}=\sqrt{8}\left(\rho^{3}-\rho \right)\sin\theta$, $Z_{8}=\sqrt{8}\left(\rho^{2}-\rho \right)\cos\theta$	Trefoil

**Table 4:** RMSE under different numbers of training data.

Number	5000	6000	8000	12,000	18,000
RMSE	0.0711±0.0953λ	0.0578±0.0830λ	0.0365±0.0600λ	0.0201±0.0392λ	0.0109±0.0241λ

## Data Availability

Data are contained within the article.

## References

[1] Ko J., Davis C.C. (2017). Comparison of the plenoptic sensor and the Shack–Hartmann sensor. Appl. Opt..

[2] Zha J., Xiao F., Kang J., Zhao H., Dai Y., Zhang Y. (2017). Statistical analysis of ocular monochromatic aberrations in Chinese population for adaptive optics op hthalmoscope design. J. Innov. Opt. Health Sci..

[3] Yoon G., Pantanelli S., Nagy L.J. (2006). Large-dynamic-range Shack-Hartmann wavefront sensor for highly aberrated eyes. J. Biomed. Opt..

[4] Cubalchini R. (1979). Modal wave-front estimation from phase derivative measurements. J. Opt. Soc. Am..

[5] Hudgin R.H. (1977). Wave-front reconstruction for compensated imaging. J. Opt. Soc. Am..

[6] Fried D.L. (1977). Least-square fitting a wave-front distortion estimate to an array of phase-difference measurements. J. Opt. Soc. Am..

[7] Southwell W. (1980). Wave-front estimation from wave-front slope measurements. J. Opt. Soc. Am..

[8] Pfund J., Lindlein N., Schwider J. (1998). Dynamic range expansion of a Shack–Hartmann sensor by use of a modified unwrapping algorithm. Opt. Lett..

[9] Roggemann M.C., Schulz T.J. (1998). Algorithm to increase the largest aberration that can be reconstructed from Hartmann sensor measurements. Appl. Opt..

[10] Groening S., Sick B., Donner K., Pfund J., Lindlein N., Schwider J. (2000). Wave-front reconstruction with a Shack–Hartmann sensor with an iterative spline fitting method. Appl. Opt..

[11] Lee J., Shack R.V., Descour M.R. (2005). Sorting method to extend the dynamic range of the Shack–Hartmann wave-front sensor. Appl. Opt..

[12] Smith D.G., Greivenkamp J.E. (2008). Generalized method for sorting Shack-Hartmann spot patterns using local similarity. Appl. Opt..

[13] Leroux C., Dainty C. (2009). A simple and robust method to extend the dynamic range of an aberrometer. Opt. Express.

[14] Vargas J., Restrepo R., Belenguer T. (2014). Shack-Hartmann spot dislocation map determination using an optical flow method. Opt. Express.

[15] Yu L., Xia M., Xie H., Xuan L., Ma J. (2014). Novel methods to improve the measurement accuracy and the dynamic range of Shack-Hartmann wavefront sensor. J. Mod. Opt..

[16] Gao Z., Li X., Ye H. (2019). Large dynamic range Shack–Hartmann wavefront measurement based on image segmentation and a neighbouring-region search algorithm. Opt. Commun..

[17] Chen H., Zhang Y., Bao H., Li L., Wei K. (2022). Hartmanngram structural information-assisted aberration measurement for a 4-meter-thin primary mirror with a large dynamic range. Opt. Commun..

[18] Yang W., Wang J., Wang B. (2022). A Method Used to Improve the Dynamic Range of Shack-Hartmann Wavefront Sensor in Presence of Large Aberration. Sensors.

[19] Lindlein N., Pfund J., Schwider J. (2000). Expansion of the dynamic range of a Shack-Hartmann sensor by using astigmatic microlenses. Opt. Eng..

[20] Lindlein N., Pfund J. (2002). Experimental results for expanding the dynamic range of a Shack-Hartmann sensor by using astigmatic microlenses. Opt. Eng..

[21] Lindlein N., Pfund J., Schwider J. (2001). Algorithm for expanding the dynamic range of a Shack-Hartmann sensor by using a spatial light modulator. Opt. Eng..

[22] Ares M., Royo S., Caum J. (2007). Shack-Hartmann sensor based on a cylindrical microlens array. Opt. Lett..

[23] Saita Y., Shinto H., Nomura T. (2015). Holographic Shack-Hartmann wavefront sensor based on the correlation peak displacement detection method for wavefront sensing with large dynamic range. Optica.

[24] Shinto H., Saita Y., Nomura T. (2016). Shack–Hartmann wavefront sensor with large dynamic range by adaptive spot search method. Appl. Opt..

[25] Aftab M., Choi H., Liang R., Kim D.W. (2018). Adaptive Shack-Hartmann wavefront sensor accommodating large wavefront variations. Opt. Express.

[26] Guo H., Korablinova N., Ren Q., Bille J. (2006). Wavefront reconstruction with artificial neural networks. Opt. Express.

[27] Li Z., Li X. (2018). Centroid computation for Shack-Hartmann wavefront sensor in extreme situations based on artificial neural networks. Opt. Express.

[28] Swanson R., Lamb M., Correia C., Sivanandam S., Kutulakos K. (2018). Wavefront reconstruction and prediction with convolutional neural networks. Adapt. Opt. Syst..

[29] Hu L., Hu S., Gong W., Si K. (2020). Deep learning assisted Shack–Hartmann wavefront sensor for direct wavefront detection. Opt. Lett..

[30] Zhang Z., Liu Q., Wang Y. (2018). Road extraction by deep residual u-net. IEEE Geosci. Remote Sens. Lett..

[31] Guo Y., Wu Y., Li Y., Rao X., Rao C. (2022). Deep phase retrieval for astronomical Shack–Hartmann wavefront sensors. Mon. Not. R. Astron. Soc..

[32] Roddier N.A. (1990). Atmospheric wavefront simulation using Zernike polynomials. Opt. Eng..

[33] Liu Z., Mao H., Wu C.Y., Feichtenhofer C., Darrell T., Xie S. A ConvNet for the 2020s. Proceedings of the IEEE/CVF Conference on Computer Vision and Pattern Recognition (CVPR).

[34] He K., Zhang X., Ren S., Sun J. Deep Residual Learning for Image Recognition. Proceedings of the IEEE Conference on Computer Vision and Pattern Recognition (CVPR).

[35] Ioannou Y., Robertson D., Cipolla R., Criminisi A. Deep Roots: Improving CNN Efficiency With Hierarchical Filter Groups. Proceedings of the IEEE Conference on Computer Vision and Pattern Recognition (CVPR).

[36] Zhao J., Xiao F., Kang J., Zhao H., Dai Y., Zhang Y. (2016). Quantifying intraocular scatter with near diffraction-limited double-pass point spread function. Biomed. Opt. Express.

[37] Zhao J., Xiao F., Zhao H., Dai Y., Zhang Y. (2017). Effect of higher-order aberrations and intraocular scatter on contrast sensitivity measured with a single instrument. Biomed. Opt. Express.

[38] Fei X. (2015). High-Resolution Adaptive Optics Retinal Microscopic Imaging with Dual Deformable Mirrors. Ph.D. Thesis.

[39] Thibos L.N., Bradley A., Hong X. (2002). A statistical model of the aberration structure of normal, well-corrected eyes. Ophthalmic Physiol. Opt..

[40] Born M., Wolf E. (1975). Principles of Optics.

